# Supplementary material for: Structural and electrophysiological basis for the modulation of KCNQ1 channel currents by ML277
Source: Nat Commun. 2022 Jun 29;13:3760. doi: 10.1038/s41467-022-31526-7 (PMC9243137; doi:10.1038/s41467-022-31526-7)
Supplement: Supplementary file 1 — Supplementary Information [file 41467_2022_31526_MOESM1_ESM.pdf]

Structural and electrophysiological basis for the modulation of KCNQ1 channel currents by ML277

Katrien Willegems, Jodene Eldstrom, Efthimios Kyriakis, Fariba Ataei, Harutyun Sahakyan, Ying Dou, Sophia Russo, Filip Van Petegem, and David Fedida

Supplementary Figures 1-10 and

Supplementary Tables 1-4

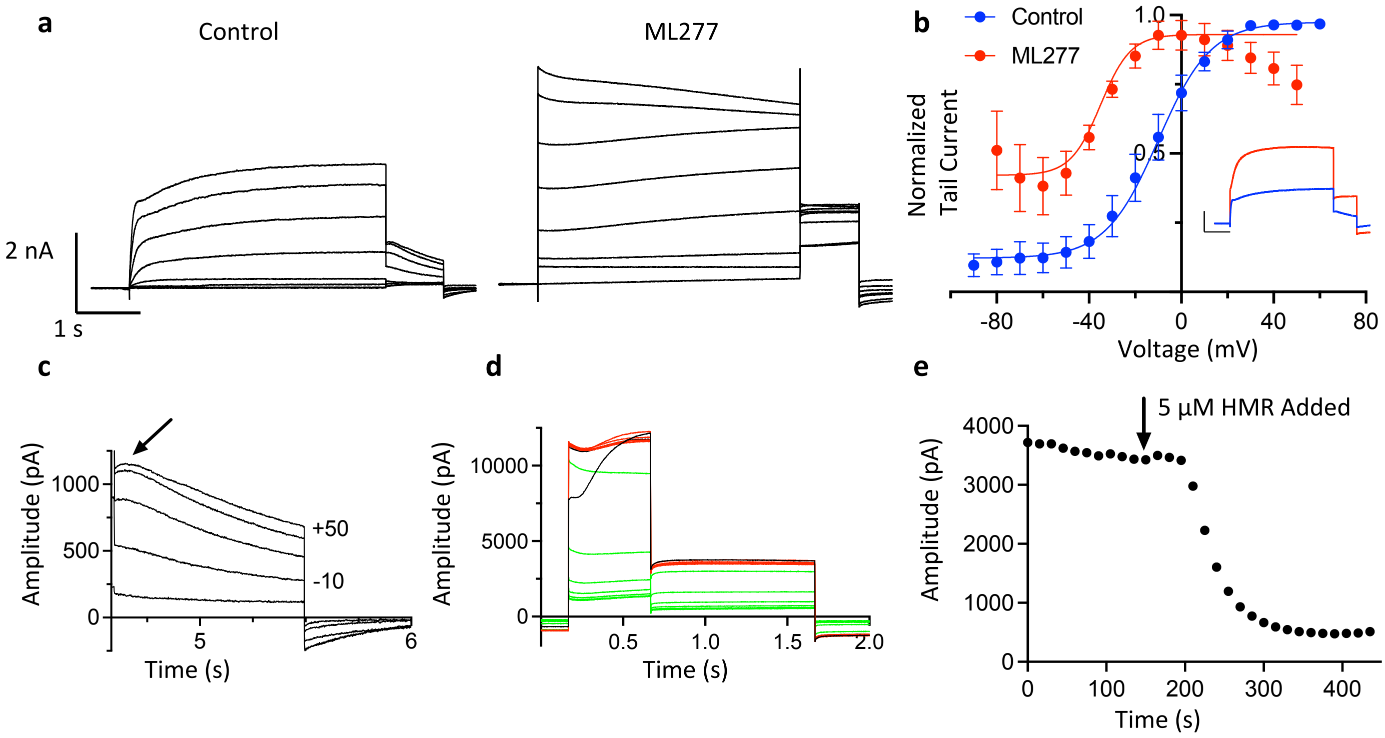

Supplementary Fig. 1. The *Xenopus* construct used for cryo-EM expresses KCNQ1 with normal kinetics and is responsive to 1 µM ML277

(a) Control xKCNQ1 currents, and in 1 µM ML277. Cells were held at -90 mV, pulsed from -90 or -80 to +60 mV in 10 mV steps for 4 s, then to -40 mV for 0.9 s. The interpulse interval was 15 s. Every other voltage is omitted for clarity. Note increase in pulse and tail current, and slowing of tail current decay caused by ML277, almost identical to effect seen on KCNQ1 (Fig. 7, Supplementary Fig. 9).

(b) G-V relationships for the xKCNQ1 channel cryo-EM construct currents expressed in tsA201 cells in control and the presence of 1 µM ML277. Protocol as in (a). Tail currents were measured immediately upon repolarization to -40 mV and normalization was performed to the peak value during the step to -40 mV for each cell. Error bars denote mean ± SEM, n = 4 cells for control and 5 cells for ML277. The $V_{1/2}$ was -9.8 ± 4.9 mV in control and -41.2 ± 4.0 mV in ML277, source data are provided as a Source Data file. Inset panel shows single current tracings in control (blue) and ML277 (red) during pulses to +60 mV, and repolarization to -40 mV. Calibration bars are 2 nA and 1 s. The mean increase in xKCNQ1 tail currents on ML277 treatment was 3.8 ± 0.9 (SEM), n = 3 cells.

(c) Tail currents recorded at -40 mV from the cryo-EM xQ1 construct, shown on an expanded time scale to show the hook in the tail currents after prepulses to -10 mV or more positive potentials that indicates inactivation is taking place during the test pulse. Tails were recorded using the same protocol as in (a).

(d) Effect of 5 µM HMR1556 on xKCNQ1 currents during ML277 exposure. Pulses were to +60 mV for 0.5 s, with a repolarizing step to -40 mV for 1 s, applied every 15 s. Black trace is the first trace (shows effect of longer time at holding potential), red traces are control ML277 and green 1 min (and every 15 s subsequently) after HMR1556 added to bath. Note that tail currents decrease and are blocked completely in the presence of HMR1556.

(e) Diary plot of tail currents from data in (d).

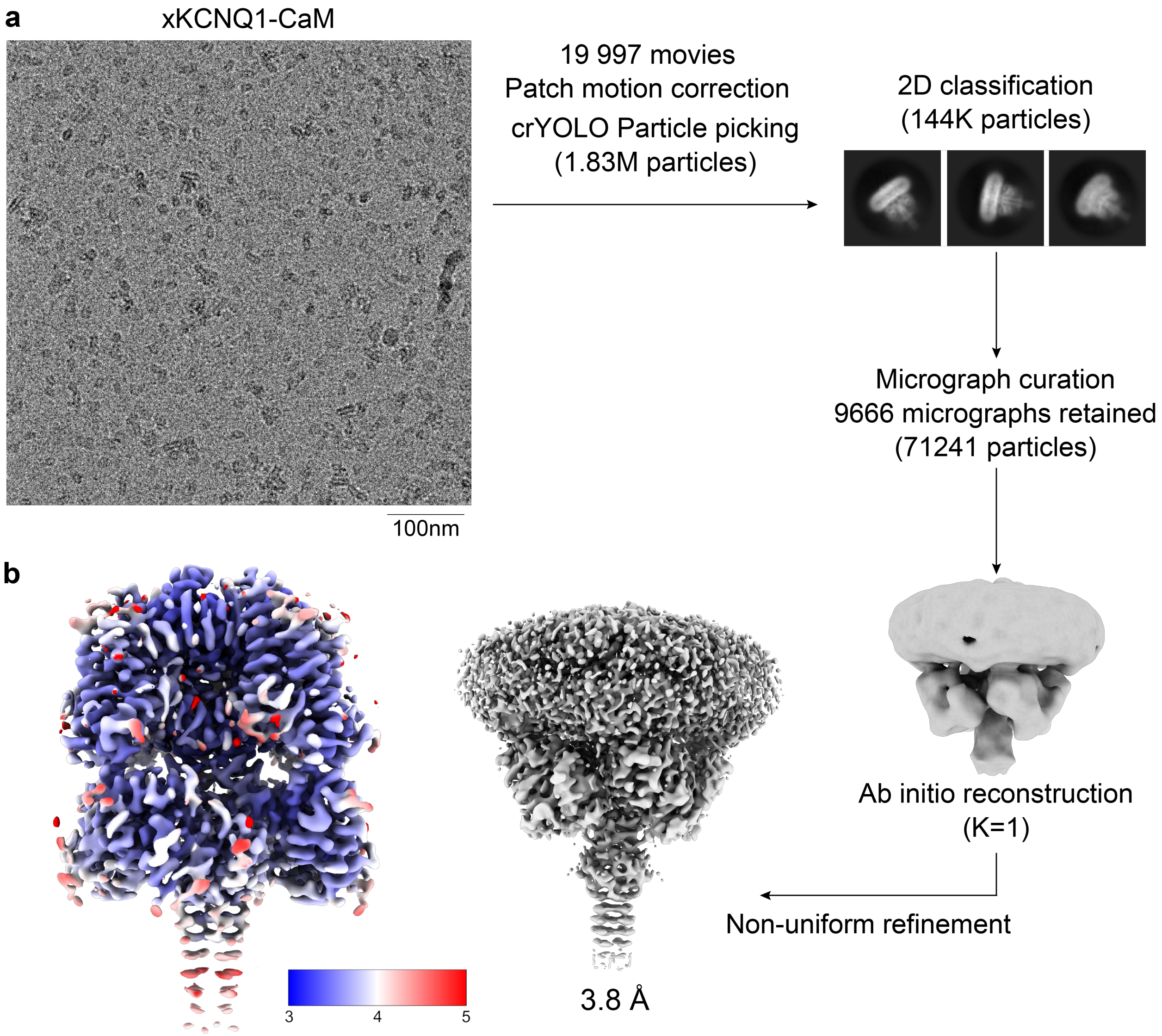

Supplementary Fig. 2. Structure determination of xKCNQ1-CaM

(a) A representative micrograph for the xKCNQ1-CaM data set and the processing pipeline for structure determination carried out in cryoSPARC with the exception of the particle picking done in crYOLO, showing 2D classes and 3D reconstructions. Details of the processing strategy are described in the Methods section.

(b) Local resolution of the xKCNQ1-CaM cryo-EM density map calculated in cryoSPARC. Scale represents 3-5 Å.

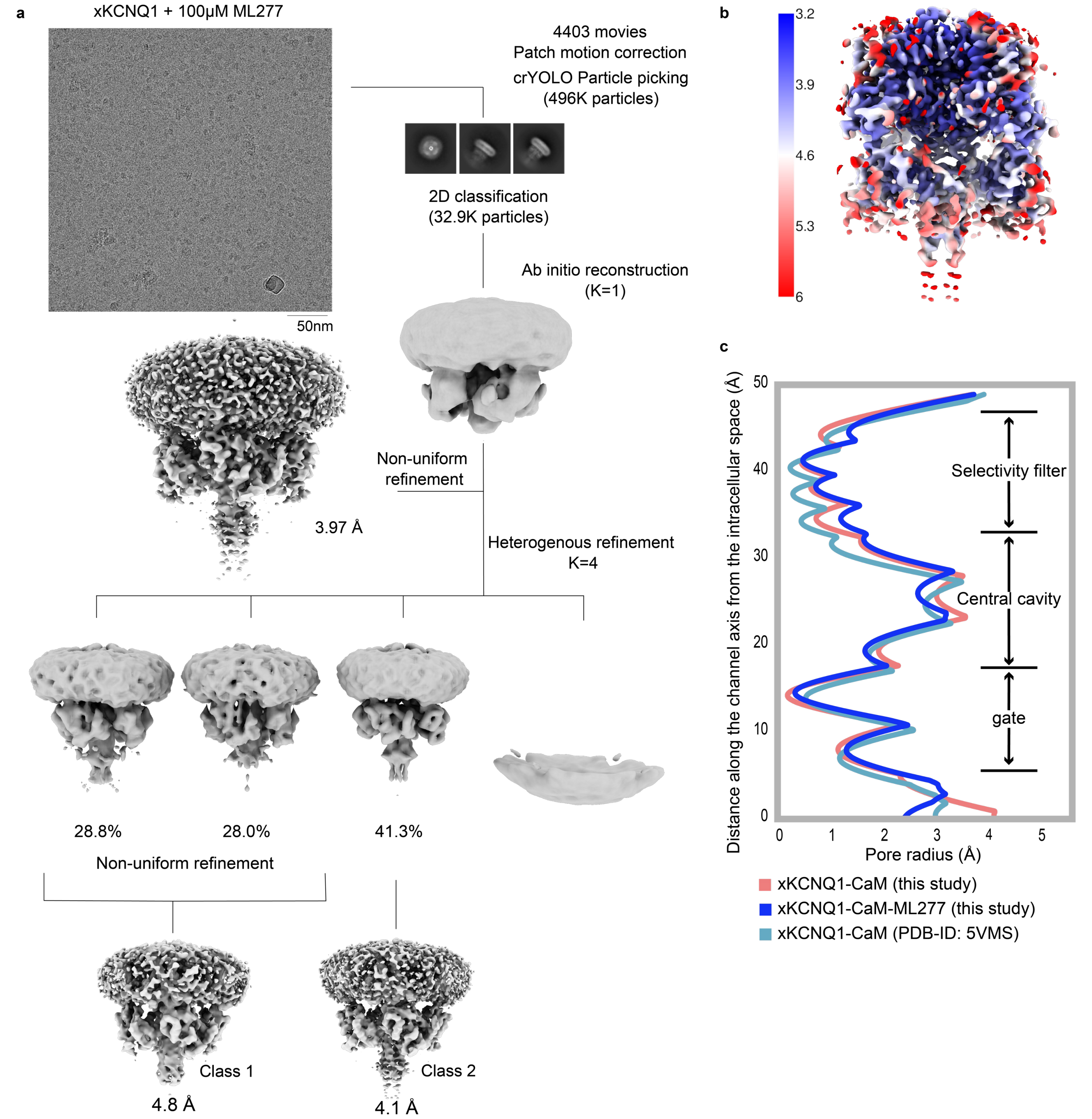

Supplementary Fig. 3. Structure determination of xKCNQ1-CaM-ML277 (data set 1)

(a) Representative micrograph for the xKCNQ1-CaM-ML277 data set 1 and the processing pipeline for structure determination carried out in cryoSPARC with the exception of the particle picking done in crYOLO, showing 2D classes and 3D reconstructions. Details of the processing strategy are described in the Material and methods sections.

(b) Local resolution of the xKCNQ1-CaM-ML277 cryo-EM density map calculated in cryoSPARC. Scale represents 3.2-6.0 Å.

(c) Pore radius along the channel axis calculated with HOLE[1]. xKCNQ1-CaM, xKCNQ1-CaM-ML277 and xKCNQ1-CaM (PDB-ID: 5VMS[2]) are in a similar closed conformation. Scale is in Å.

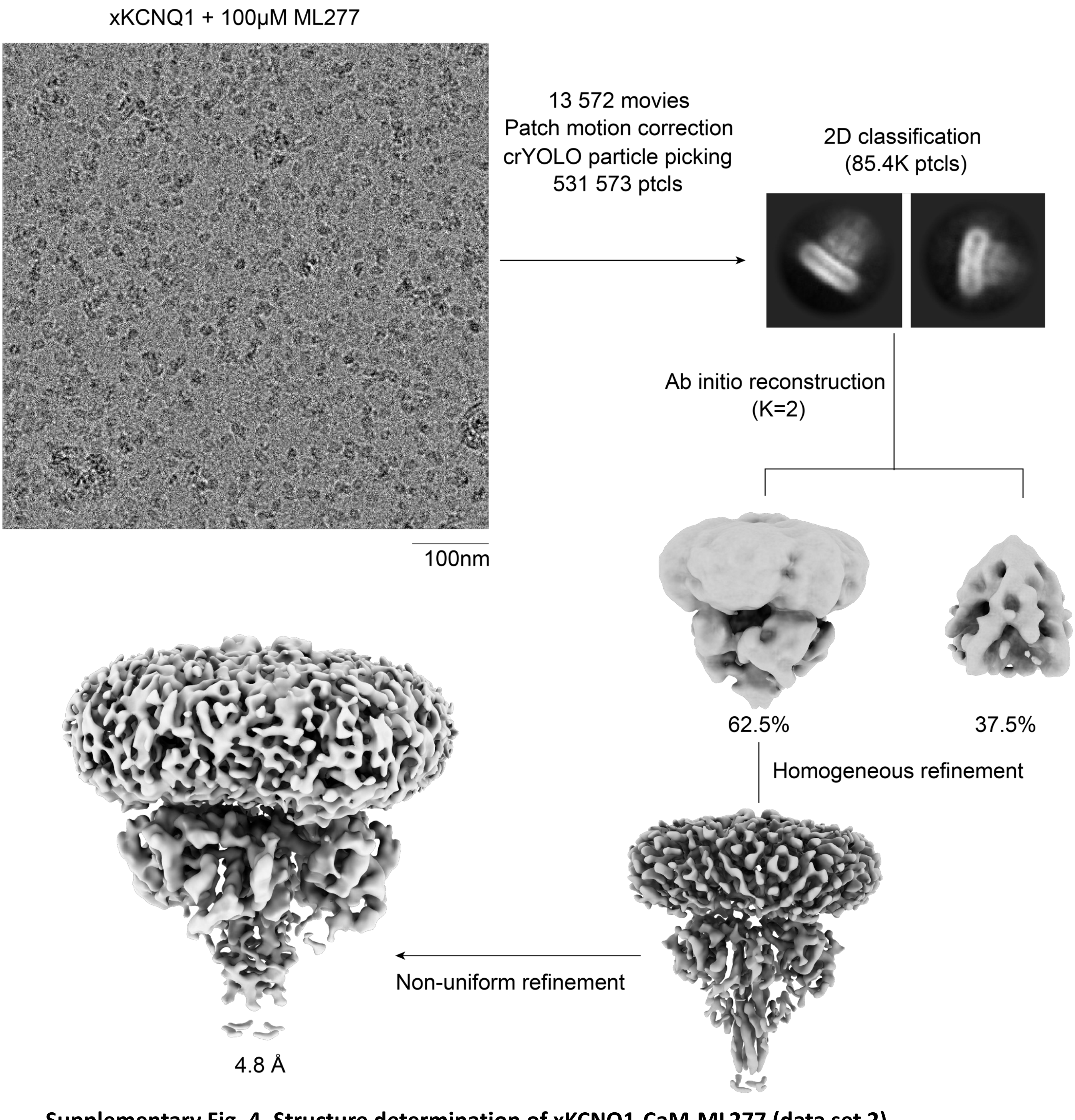

Supplementary Fig. 4. Structure determination of xKCNQ1-CaM-ML277 (data set 2)
Representative micrograph and the pipeline of xKCNQ1-CaM-ML277 structure determination for the second data set carried out in cryoSPARC with the exception of particle picking done in crYOLO.

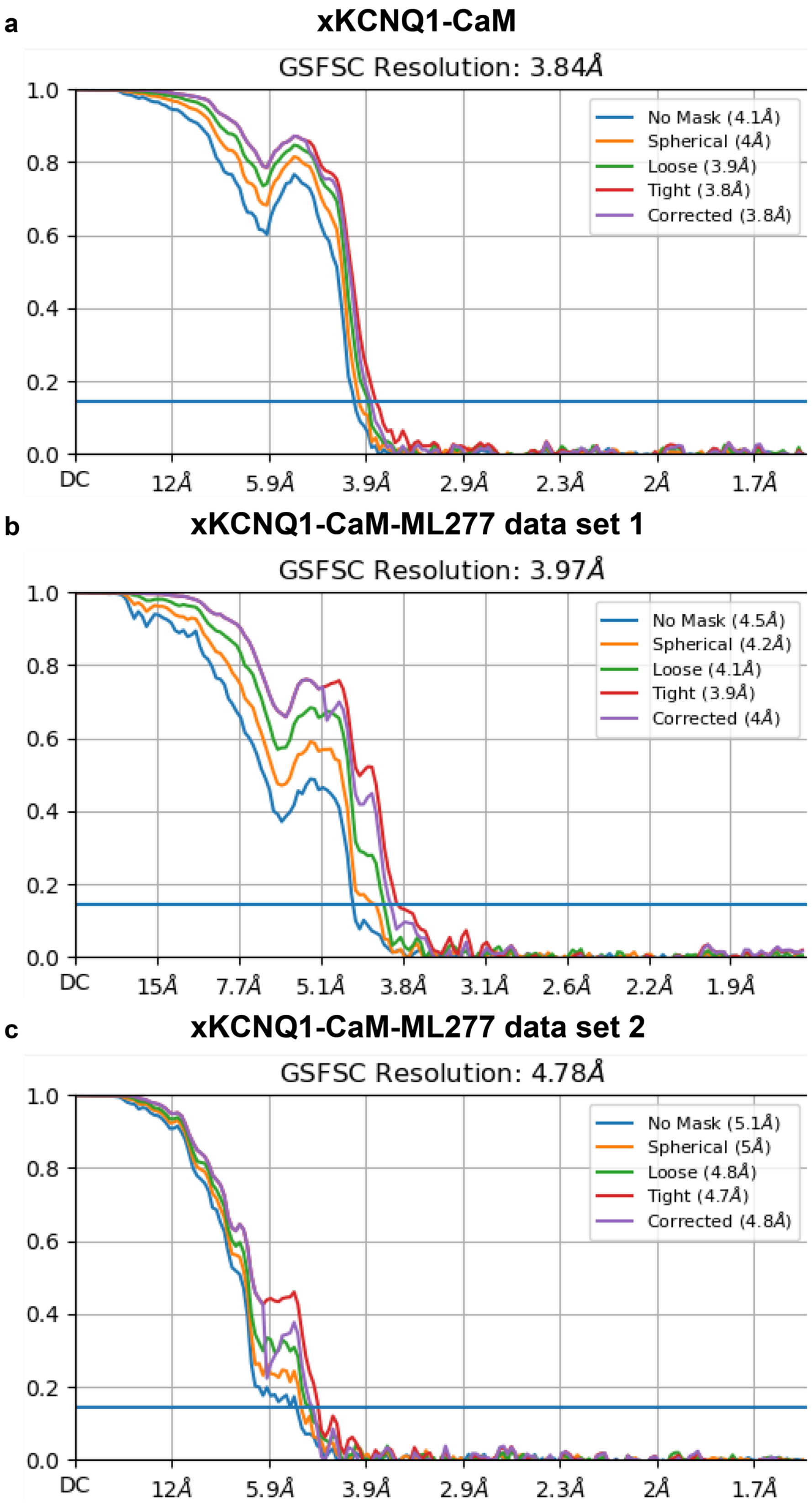

Supplementary Fig. 5. Gold standard Fourier shell correlation (FSC) curves as generated by cryoSPARC

(a) FSC curves of final maps for xKCNQ1-CaM.

(b) xKCNQ1-CaM-ML277 data set 1.

(c) xKCNQ1-CaM-ML277 data set 2

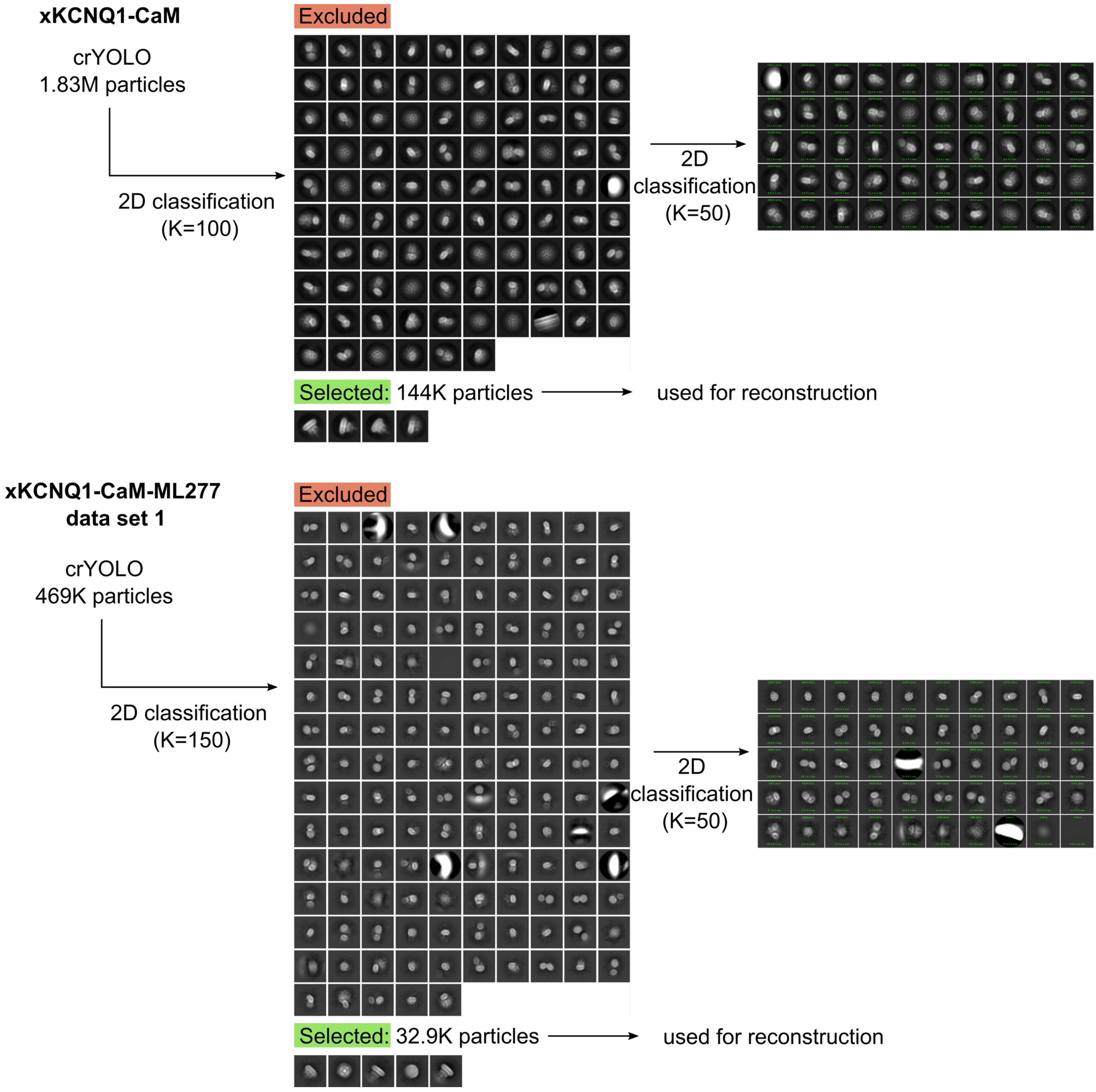

Supplementary Fig. 6. Exploration of excluded 2D classes

For the xKCNQ1-CaM dataset (above) and xKCNQ1-CaM-ML277 data set 1 (below), particles from classes resembling 2D projections of KCNQ were used for 3D reconstructions. Particles from excluded classes were used for an additional round of 2D classification (K=50) and showed no classes representing KCNQ projections.

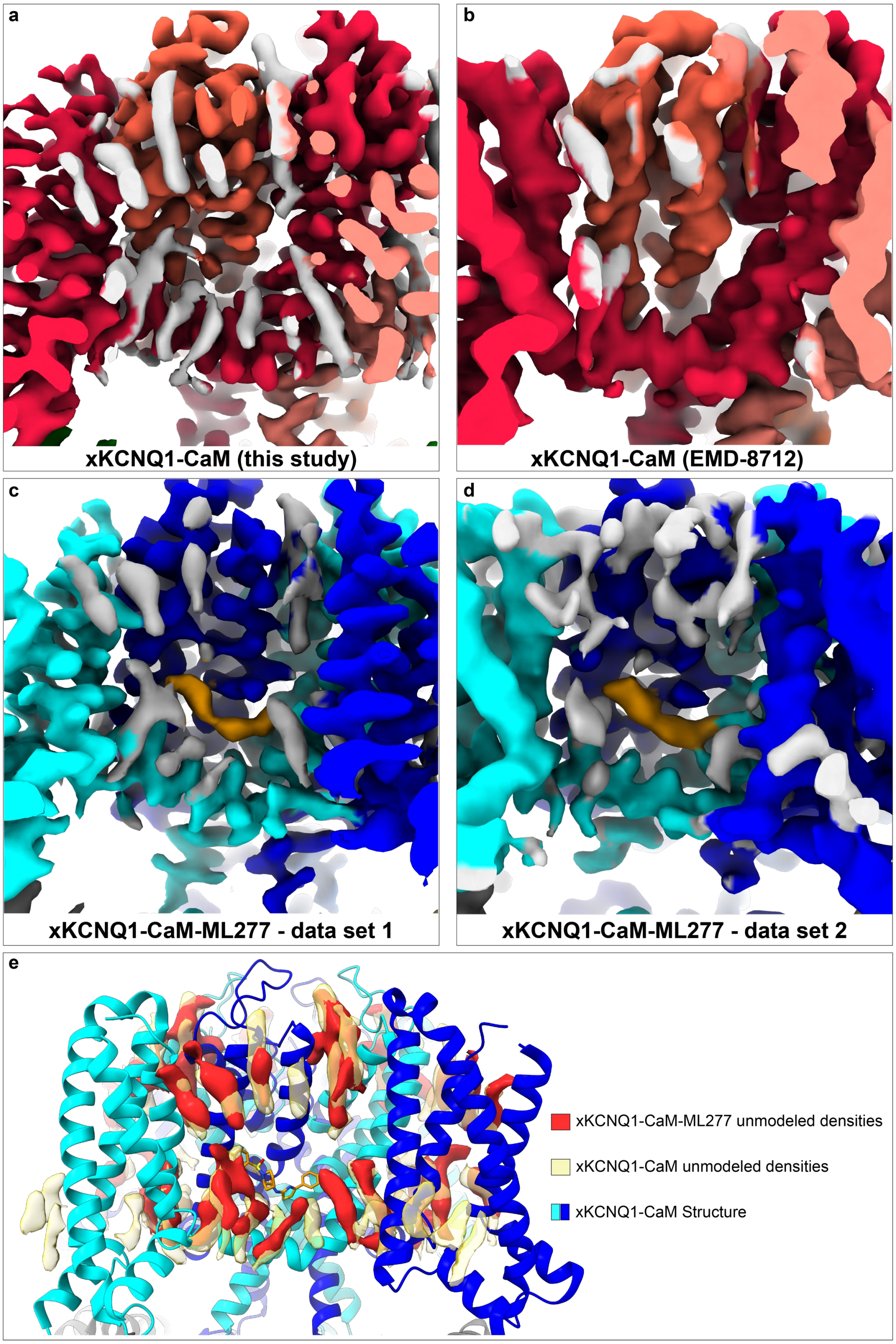

Supplementary Fig. 7. Comparison of the TM region of two independent xKCNQ1-CaM and two independent xKCNQ1-CaM-ML277 data sets

(a-d) Focus on TM region corresponding to potential ML277 binding sites in two xKCNQ1-CaM (a, b; this study and EMD-8712[2]) and two xKCNQ1-CaM-ML277 (c, d; this study) cryo-EM density maps. The additional density corresponding to ML277 is colored orange and unmodeled densities, likely corresponding to detergent molecules, are indicated in grey. (e) Superposition of the unmodeled densities onto xKCNQ1-CaM-ML277 structure shows that there is no other unique density in the xKCNQ1-CaM-ML277 map that could fit the elongated shape of ML277.

ChimeraX ('color zone') was used for coloring the density based on the fitted models with a cut off distance for coloring set at 3.5 Å, leading to partial coloring of unmodeled densities in some panels.

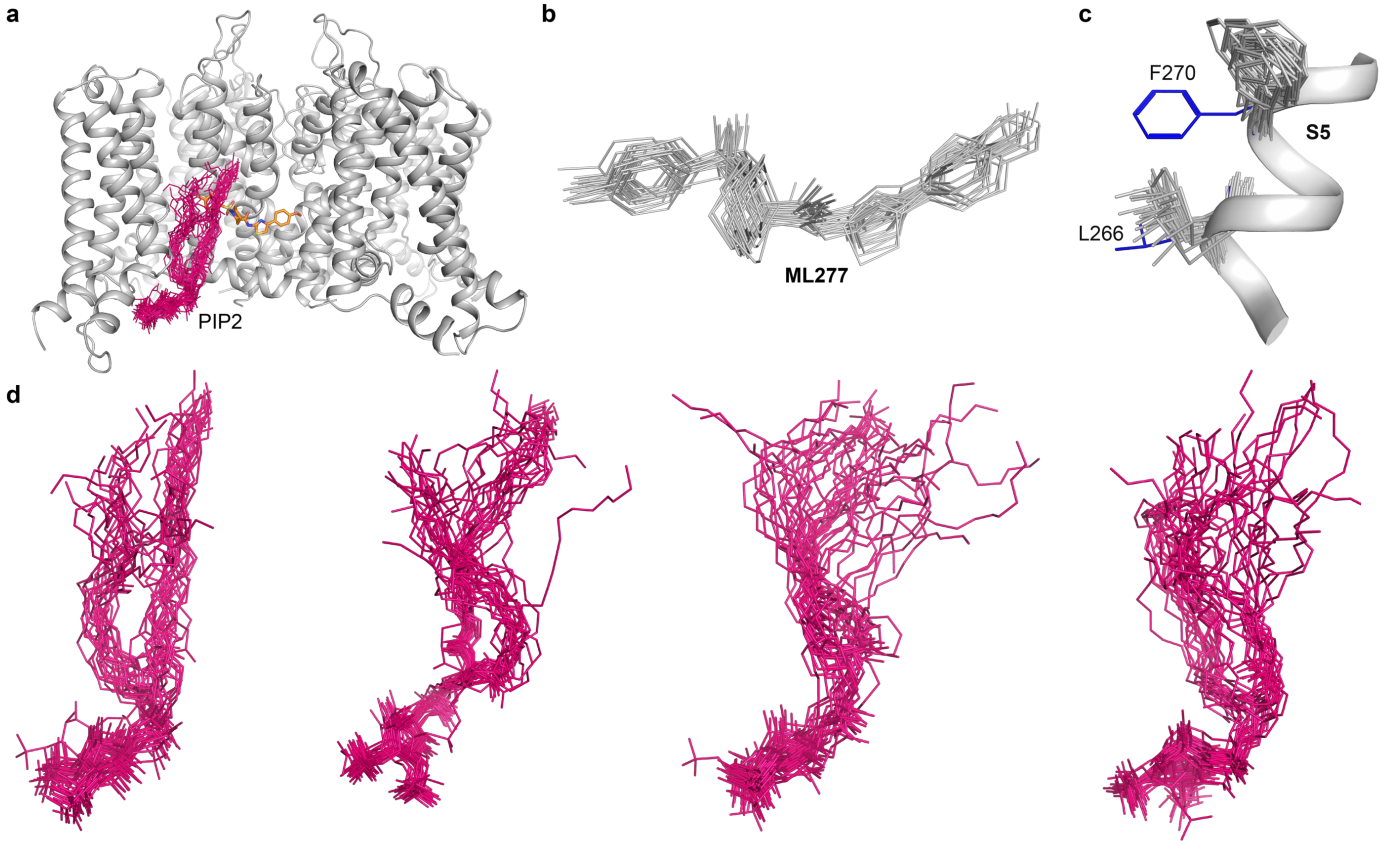

Supplementary Fig. 8. MD simulations of ML277 in the presence of PIP2.

a) Superposition of PIP2 coordinates from MD simulation. ML277 is bound to the open pore channel hKCNQ1-KCNE3-PIP2 (PDB-ID: 6V01[3]), with KCNE3 removed.

b) Superposition of ML277 coordinates from MD simulations.

c) Re-orientation of F270 and L266 in hKCNQ1-PIP2-ML277 during MD simulation. Note that sidechain orientation now matches that seen in the cryo-EM xKCNQ1-CaM structure (Fig.2, Fig.4). Initial positions for these residues in hKCNQ1-CaM-KCNE3-PIP2 structure are shown in blue.

d) Superposition of 80 poses (20 in each of the sub-panels) of PIP2 highlights the flexibility of the lipid tail.

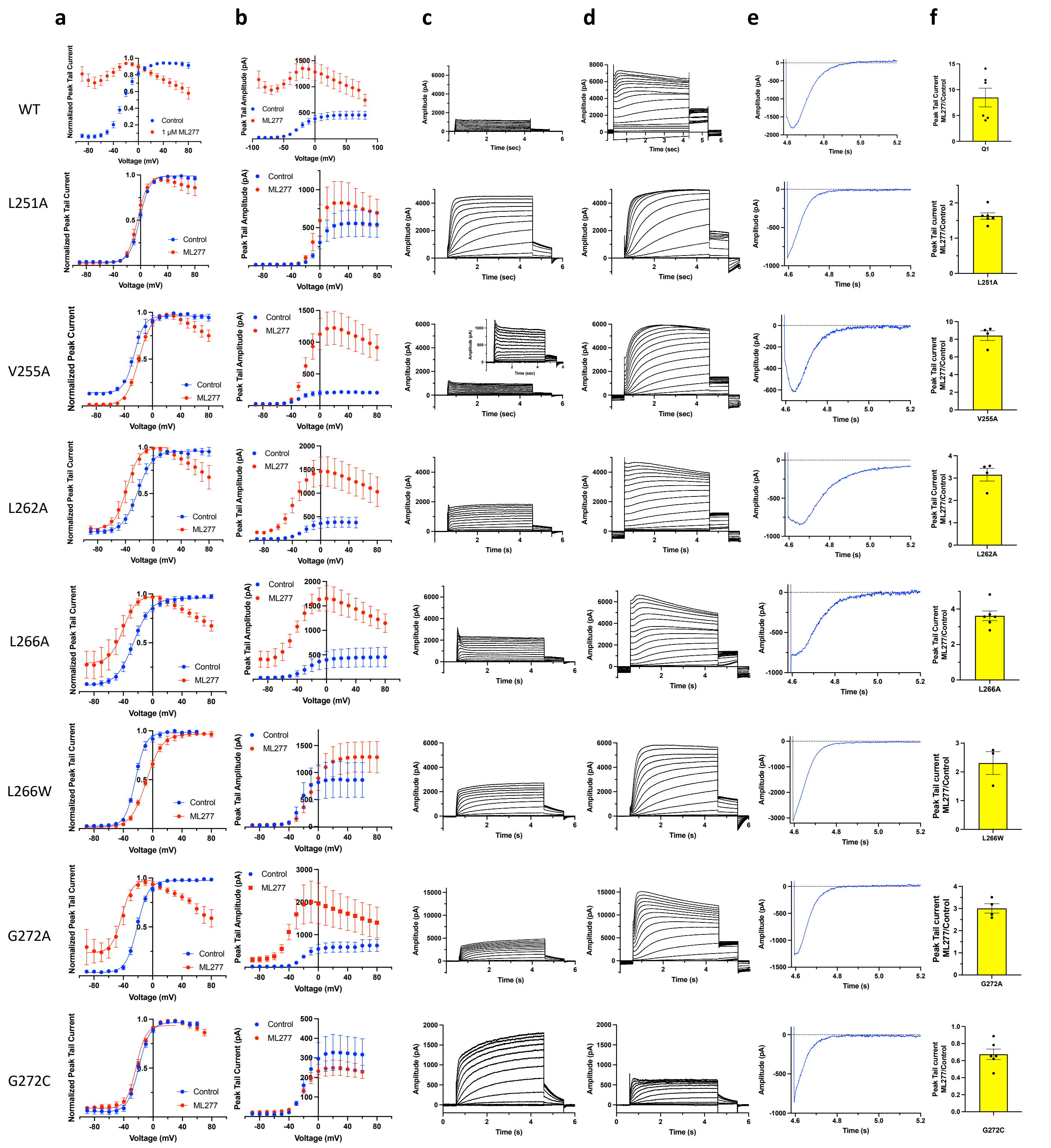

Supplementary Fig. 9. Compiled electrophysiological data from WT and ML277 binding site mutants

(a and b) Normalized and un-normalized peak tail current-voltage (G-V) relationships in control (blue) and in 1 μM ML277 (red). Data are shown mean ± SEM, n = 3-17 cells (see Supplementary Table 3 for exact n values). (c and d) Current traces in control (c) and after addition of 1 μM ML277 (d). Cells were held at -90 mV, pulsed to +60 mV for 4 s, then -40 mV for 0.9 s. The interpulse interval was 15 s. (e) Tail currents obtained at -120 mV after 4 s pulses to +60 mV in the absence of ML277 for different mutants as indicated. (f) KCNQ1 tail current amplitudes in ML277 divided by initial control current, protocol as in A. Error bars denote mean ± SEM. Individual measures are shown, n = 3-8 cells, (see Supplementary Table 2 for exact n values). Source data are provided as a Source Data file.

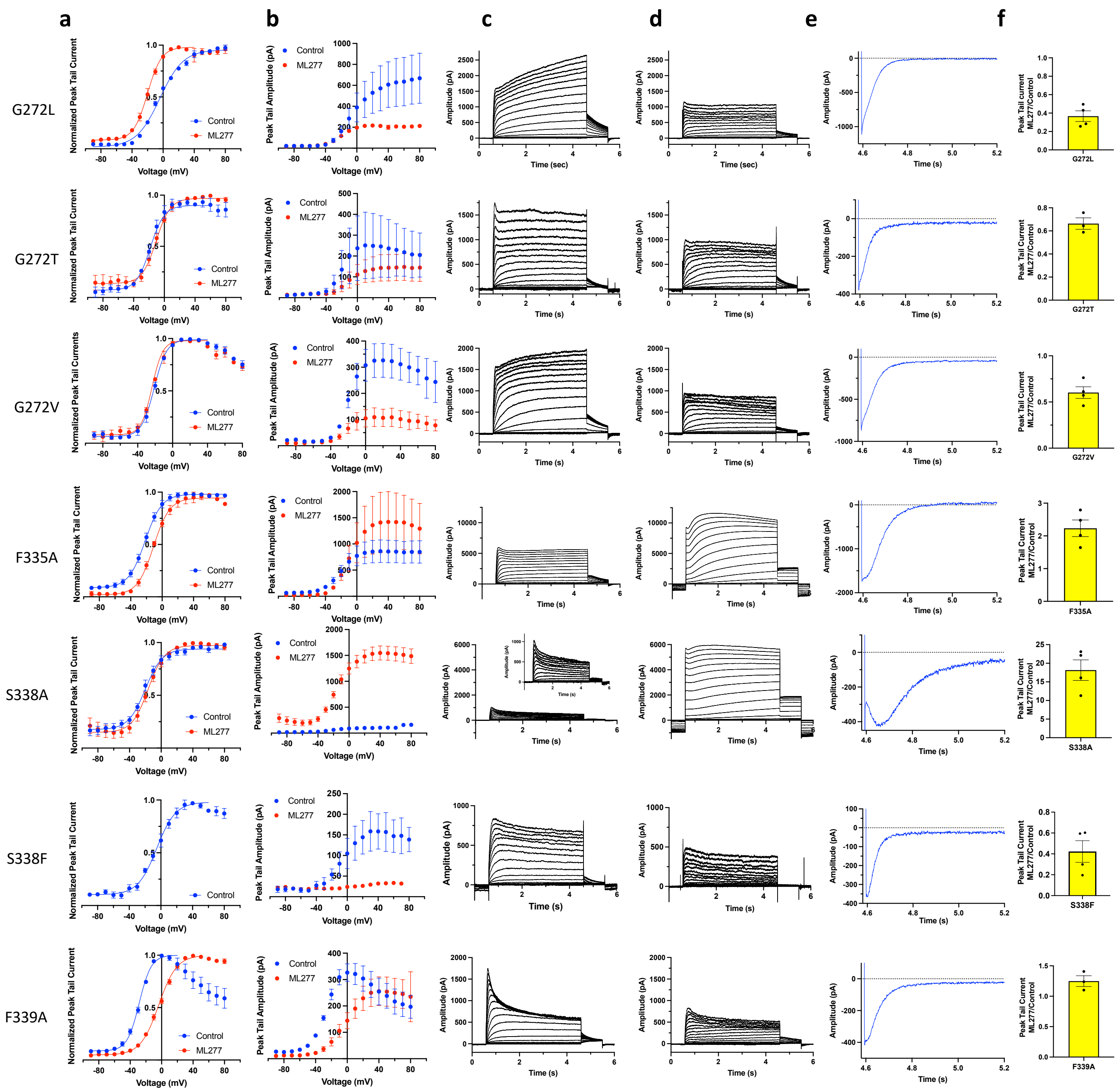

Supplementary Fig. 9, continued. Compiled electrophysiological data from WT and ML277 binding site mutants (a and b) Normalized and un-normalized peak tail current-voltage (G-V) relationships in control (blue) and in 1 μM ML277 (red). Data are shown mean ± SEM, n = 3-17, (see Supplementary Table 3 for exact n values). (c and d) Current traces in control (c) and after addition of 1 μM ML277 (d). Cells were held at -90 mV, pulsed to +60 mV for 4 s, then -40 mV for 0.9 s. The interpulse interval was 15 s. (e) Tail currents obtained at -120 mV after 4 s pulses to +60 mV in the absence of ML277 for different mutants as indicated. (f) KCNQ1 tail current amplitudes in ML277 divided by initial control current, protocol as in A. Error bars denote mean ± SEM. Individual measures are shown, n = 3-8 (see Supplementary Table 2 for exact n values). Source data are provided as a Source Data file.

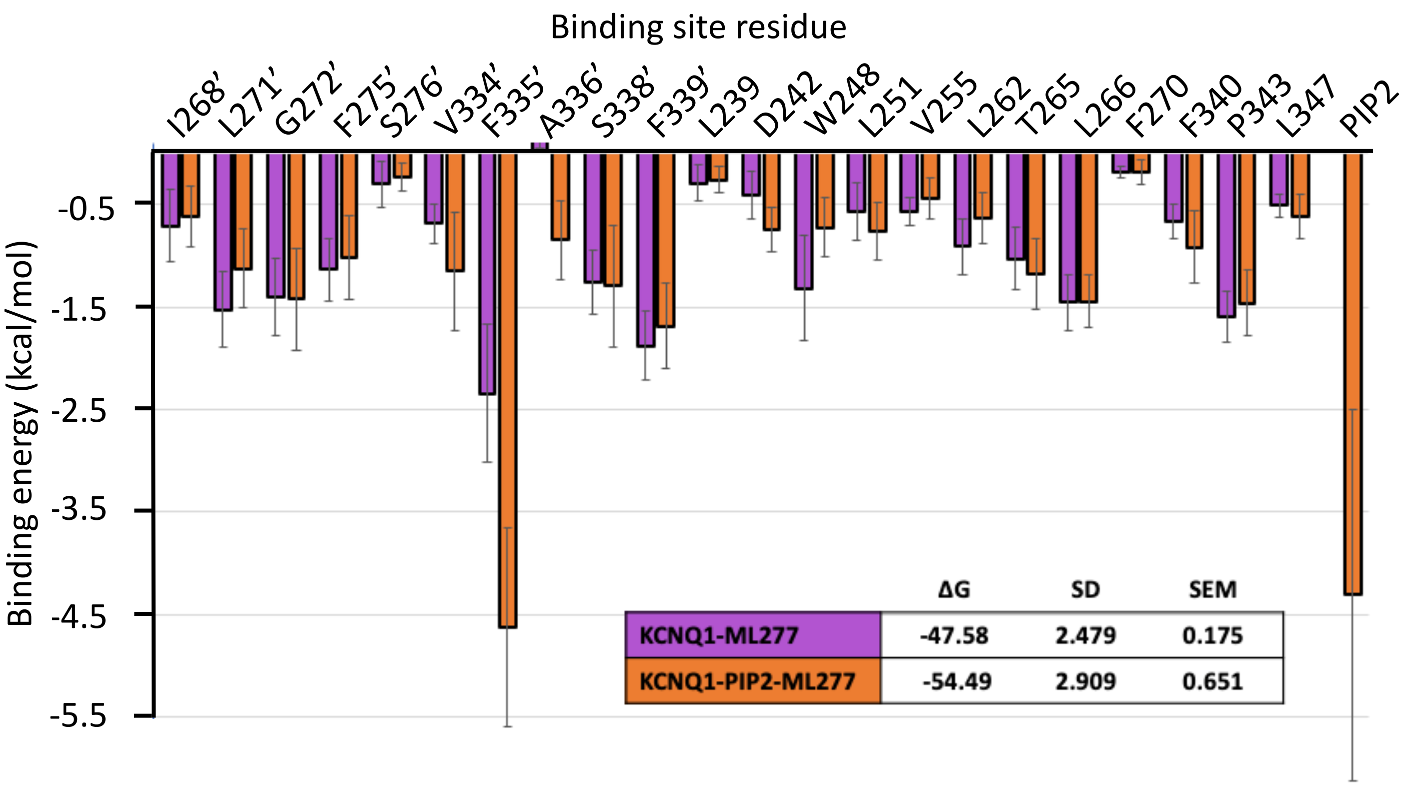

Supplementary Fig. 10. Binding free energy per-residue decomposition.
Binding energies of residues interacting with ML277 in hKCNQ1-ML277 and hKCNQ1-PIP2-ML277 complexes calculated with the MM/PBSA method. Residues from two subunits comprising the binding pocket are listed above, with the apostrophe denoting residues in the second protomer. Mean binding energy per residue is shown by the horizontal bar line, above and below which error bars show SD of the binding energy during MD simulation. The SD and SEM are of energy fluctuations between the 200 snapshots used (see Free Energy calculations in Methods).

	xKCNQ1	hKCNQ1	hKCNQ2	KCNQ3	hKCNQ4	hKCNQ5
**S4-S5 Linker**	Trp238	Trp248	Trp218	Trp247	Trp224	Trp252
	Leu241	Leu251	Leu221	Leu250	Leu227	Leu255
	Val245	Val255	Val225	Ile254	Val231	Val259
**S5**	Leu252	Leu262	Leu232	Leu261	Leu238	Leu266
	Thr255	Thr265	Ala235	Ala264	Ala241	Ala269
	Leu256	Leu266	Trp236	Trp265	Trp242	Trp270
	Phe260	Phe270	Phe240	Phe269	Phe246	Phe274
**S6**	Pro333	Pro343	Pro308	Pro347	Pro314	Pro342
	Leu337	Leu347	Leu312	Leu351	Leu318	Leu346
**S5’**	Ile258	Ile268	Ile238	Ile267	Ile244	Ile272
	Leu261	Leu271	Leu241	Leu270	Leu247	Leu275
	Gly262	Gly272	Cys242	Thr271	Val248	Val276
	Phe265	Phe275	Leu245	Leu274	Phe251	Phe279
**S6’**	Phe322	Phe332	Phe297	Phe336	Phe303	Phe331
	Val324	Val334	Leu299	Leu338	Leu305	Leu333
	Phe325	Phe335	Ile300	Ile339	Leu306	Leu334
	Ala326	Ala336	Gly301	Gly340	Gly307	Gly335
	Ser328	Ser338	Ser303	Ser342	Ser309	Ser337
	Phe329	Phe339	Phe304	Phe343	Phe310	Phe338

Supplementary Table 1. Residues in the xKCNQ1-CaM ML277-binding site compared with the homologous residues in other KCNQ isoforms. Residues that differ are indicated in red. The apostrophe denotes residues in the second subunit protomer.

	Control		ML277		
	Mean ± SEM	n	Mean ± SEM	n	P value
**WT**	320 ± 61	8	2116 ± 274	8	p=0.0002
**L251A**	467 ± 152	6	752 ± 246	6	p=0.0328
**V255A**	181 ± 20	4	1501± 151	4	p=0.0024
**L262A**	403 ± 83	4	1298 ± 338	6	p=0.0409
**L266A**	427 ± 70	6	1483 ± 215	6	p=0.0009
**L266W**	531 ± 189	5	1122 ± 302	5	p=0.0210
**G272A**	678 ± 142	5	1873 ± 378	5	p=0.0083
**G272C**	296 ± 49	6	197 ± 37	6	p=0.0101
**G272T**	132 ± 28	4	86 ± 25	4	p=0.0032
**G272V**	231 ± 54	4	132 ± 28	4	p=0.0421
**G272L**	602 ± 217	4	220 ± 67	4	ns
**F335A**	768 ± 187	4	1715 ± 432	4	p=0.0405
**S338A**	121 ± 31	5	1665 ± 234	5	p=0.0020
**S338F**	147 ± 32	5	59.0 ± 12	5	p=0.0239
**F339A**	221 ± 47	3	273 ± 51	3	ns

Supplementary Table 2: Mean tail current amplitudes before and after 1 μM ML277. Cells were held at -90 mV and pulsed to +60 mV for 4 s, then to -40 mV for 0.9 s, with an interpulse interval of 15 s. Statistical comparisons were performed using a two-tailed paired t-tests in GraphPad Prism, n represents individual cells.

	Control			ML277			
	$V_{1/2}$	slope	n	$V_{1/2}$	slope	n	P value
**WT***	-23.0 ± 1.7	11.9 ± 1.0	17	-	-	10	-
**L251A**	-1.3 ± 0.8	7.4 ± 0.5	5	-4.5 ± 2.0	7.5 ± 0.5	5	ns
**V255A**	-25.9 ± 3.2	9.1 ± 2.4	3	-19.5 ± 1.4	9.1 ± 0.8	4	ns†
**L262A**	-22.2 ± 2.8	10.7 ± 1.5	4	-39.4 ± 3.5	7.6 ± 0.3	4	p=0.0086
**L266A**	-25.6 ± 1.9	11.9 ± 0.8	5	-40.5 ± 3.5	9.0 ± 0.7	4	p=0.0056†
**L266W**	-23.1 ± 1.0	7.2 ± 1.0	3	-8.2 ± 0.9	11.0 ± 1.6	3	p=0.0051
**G272A**	-21.0 ± 1.1	7.8 ± 0.7	5	-42.2 ± 3.5	7.2 ± 0.4	4	p=0.0004†
**G272C**	-19.5 ± 1.2	7.7 ± 0.8	4	-23.1 ± 3.5	7.1 ± 0.9	4	ns
**G272T**	-17.4 ± 3.0	7.4 ± 1.3	3	-11.2 ± 1.0	8.9 ± 0.8	4	ns†
**G272V**	-21.1 ± 1.5	7.2 ± 0.7	4	-23.7 ± 1.3	6.2 ± 0.7	4	ns
**G272L**	-4.2 ± 0.5	15.1 ± 0.7	4	-20.2 ± 2.1	9.0 ± 0.6	3	p=0.0003†
**F335A**	-21.6 ± 1.7	10.7 ± 1.5	4	-10.9 ± 1.1	10.3 ± 0.7	3	p=0.0049†
**S338A**	-20.1 ± 3.1	10.7 ± 1.5	6	-14.3 ± 2.7	10.1 ± 0.8	5	ns†
**S338F**	-5.3 ± 2.9	11.9 ± 0.6	3	-	-	3	-
**F339A**	-28.5 ± 0.8	7.7 ± 0.3	3	-3.7 ± 1.0	11.5 ± 1.1	3	p<0.0025

Supplementary Table 3: $V_{1/2}$ of activation and slope of G-V plots

$V_{1/2}$ of activation was obtained from tail current amplitude at the start of the -40 mV step of voltage-clamp protocols (-90 mV holding potential, pulsed from -90 up to +60 to +100 mV in 10 mV steps for 4 s, then to -40 mV for 1 s, with an interpulse interval of 15 s). Statistical comparison was performed using two-tailed paired (when a before and after drug set of data was collected per cell) or unpaired t-tests (when a before or after drug GV was missing for a cell(s)) in GraphPad Prism.

* Some WT data are from Eldstrom et al., 2021[4].

† indicates an unpaired t-test was used because not all data were matched.

Data collection and processing	KCNQ1-CaM-ML277 (PDB-ID: 7TCI)	KCNQ1-CaM (PDB-ID: 7TCP)
Magnification	96,000	165,000
Voltage (kV)	300	300
Electron exposure (e–/Å²)	50	50
Defocus range (μm)	-0.5-3	-0.5-3
Pixel size (Å)	0.85	0.77
Symmetry imposed	Point, C4	Point, C4
Initial particle images (no.)	496,000	1,830,000
Final particle images (no.)	32,900	71,241
Map resolution (Å)<br>FSC threshold	 0.143	 0.143
Map resolution range (Å)	3.0-5.0	3.2-6.0
Refinement		
Initial model used (PDB code)	5VMS	5VMS
Model resolution (Å)<br>FSC threshold	3.9	3.84
Model resolution range (Å)	3.0-5.0	3.2-6.0
Map sharpening B factor (Å²)	115.7	148.7
Model composition<br>Non-hydrogen atoms<br>Protein residues<br>Ligands	 14992<br>1912<br>12	 15084<br>1916<br>16
B factors (Å²)<br>Protein<br>Ligand	 78.77<br>32.24	 5.98<br>10.22
R.M.S. deviations<br>Bond lengths (Å)<br>Bond angles (°)	 0.002<br>0.408	 0.002<br>0.365
Validation<br>MolProbity score<br>Clashscore<br>Poor rotamers (%)	 1.41<br>6.68<br>3.15	 1.78<br>3.91<br>1.33
Ramachandran plot<br>Favored (%)<br>Allowed (%)<br>Disallowed (%)	 98.04<br>1.96<br>0.00	 98.14<br>1.86<br>0.00

Supplementary Table 4: Cryo-EM data collection, refinement and validation statistics.

Supplementary References

1 Smart, O. S., Neduvelil, J. G., Wang, X., Wallace, B. A. & Sansom, M. S. HOLE: a program for the analysis of the pore dimensions of ion channel structural models. *J Mol Graph* **14**, 354-360, 376, doi:10.1016/s0263-7855(97)00009-x (1996).

2 Sun, J. & MacKinnon, R. Cryo-EM Structure of a KCNQ1/CaM Complex Reveals Insights into Congenital Long QT Syndrome. *Cell* **169**, 1042-1050 e1049, doi:10.1016/j.cell.2017.05.019 (2017).

3 Sun, J. & MacKinnon, R. Structural Basis of Human KCNQ1 Modulation and Gating. *Cell* **180**, 340-347 e349, doi:10.1016/j.cell.2019.12.003 (2020).

4 Eldstrom, J., McAfee, D. A., Dou, Y., Wang, Y. & Fedida, D. ML277 regulates KCNQ1 single-channel amplitudes and kinetics, modified by voltage sensor state. *J Gen Physiol* **153**, doi:10.1085/jgp.202112969 (2021).
