# Supplementary material for: Structural and electrophysiological basis for the modulation of KCNQ1 channel currents by ML277
Source: Nat Commun. 2022 Jun 29;13:3760. doi: 10.1038/s41467-022-31526-7 (PMC9243137; doi:10.1038/s41467-022-31526-7)
Supplement: Supplementary file 3 — Description of Additional Supplementary Files [file 41467_2022_31526_MOESM3_ESM.pdf]

File name: Supplementary Movie 1

Description: Animation of the twist of the coiled coil C-terminal domain induced by the binding of ML277 to xKCNQ1-CaM. KCNQ1 is shown in gray, one protomer is highlighted in blue, CaM has been removed for clarity and ML277 is shown in orange surface. The binding of ML277 on xKCNQ1- CaM leads to a twist of the coiled coil C-terminal domain (Fig. 3). The animation was prepared with Chimera using the Morph Conformations tool.
