# Supplementary material for: Structural and electrophysiological basis for the modulation of KCNQ1 channel currents by ML277
Source: Nat Commun. 2022 Jun 29;13:3760. doi: 10.1038/s41467-022-31526-7 (PMC9243137; doi:10.1038/s41467-022-31526-7)
Supplement: Supplementary file 5 — Reporting Summary [file 41467_2022_31526_MOESM5_ESM.pdf]

nature portfolio

Corresponding author(s): Fedida, D., and Van Petegem, F.

Last updated by author(s): May 31, 2022

Reporting Summary

Nature Portfolio wishes to improve the reproducibility of the work that we publish. This form provides structure for consistency and transparency in reporting. For further information on Nature Portfolio policies, see our Editorial Policies and the Editorial Policy Checklist.

Statistics

For all statistical analyses, confirm that the following items are present in the figure legend, table legend, main text, or Methods section.

n/a	Confirmed	
☐	☒	The exact sample size (*n*) for each experimental group/condition, given as a discrete number and unit of measurement
☐	☒	A statement on whether measurements were taken from distinct samples or whether the same sample was measured repeatedly
☐	☒	The statistical test(s) used AND whether they are one- or two-sided<br>*Only common tests should be described solely by name; describe more complex techniques in the Methods section.*
☐	☒	A description of all covariates tested
☒	☐	A description of any assumptions or corrections, such as tests of normality and adjustment for multiple comparisons
☐	☒	A full description of the statistical parameters including central tendency (e.g. means) or other basic estimates (e.g. regression coefficient) AND variation (e.g. standard deviation) or associated estimates of uncertainty (e.g. confidence intervals)
☐	☒	For null hypothesis testing, the test statistic (e.g. *F*, *t*, *r*) with confidence intervals, effect sizes, degrees of freedom and *P* value noted<br>*Give P values as exact values whenever suitable.*
☒	☐	For Bayesian analysis, information on the choice of priors and Markov chain Monte Carlo settings
☒	☐	For hierarchical and complex designs, identification of the appropriate level for tests and full reporting of outcomes
☒	☐	Estimates of effect sizes (e.g. Cohen's *d*, Pearson's *r*), indicating how they were calculated

Our web collection on statistics for biologists contains articles on many of the points above.

Software and code

Policy information about availability of computer code

Data collection	Clampex 10.1
Data analysis	Clampfit V10.1, Prizm V9, MS Excel, Coot V0.9.7, PHENIX V1.19.2-4158, UCSF ChimeraX V1.2, UCSF Chimera V1.15, Pymol V2.3.2, CrYOLO V1.7.6, Hole V2.2.005, CTFFIND V4.0, cryoSPARC V3.3.1

For manuscripts utilizing custom algorithms or software that are central to the research but not yet described in published literature, software must be made available to editors and reviewers. We strongly encourage code deposition in a community repository (e.g. GitHub). See the Nature Portfolio guidelines for submitting code & software for further information.

Data

Policy information about availability of data

All manuscripts must include a data availability statement. This statement should provide the following information, where applicable:

- Accession codes, unique identifiers, or web links for publicly available datasets
- A description of any restrictions on data availability
- For clinical datasets or third party data, please ensure that the statement adheres to our policy

Structural data that support the findings of this study have been deposited in the PDB with the deposition numbers D_1000261989, PDB-ID: 7TCI [https://doi.org/10.2210/pdb7TCI/pdb] (xKCNQ1-CaM) and D_1000262040 PDB-ID: 7TCP [https://doi.org/10.2210/pdb7TCP/pdb] (xKCNQ1-Cam In complex with ML277). Other previously published PDB files used in this manuscript were PDB-ID: 6v01 [http://doi.org/10.2210/pdb6V01/pdb] (KCNQ1-KCNE3-CaM complex with PIP2); PDB-ID: 5VMS, [http://doi.org/10.2210/pdb5VMS/pdb] (CryoEM structure of Xenopus KCNQ1 channel); PDB-ID: 7cr3, [http://doi.org/10.2210/pdb7CR3/pdb] (human KCNQ2-CaM in apo state); PDB-ID: 7byl, [http://doi.org/10.2210/pdb7BYL/pdb] (Cryo-EM structure of human KCNQ4); PDB-ID: 6UZZ, [http://doi.org/10.2210/pdb6UZZ/pdb] (human KCNQ1-CaM complex); PDB-ID: 7CR0, [http://doi.org/10.2210/pdb7CR0/pdb] (human KCNQ2 in apo state); PDB-ID: 7VNQ,

[http://doi.org/10.2210/pdb7VNQ/pdb] (human KCNQ4-ML213 complex in nanodisc). The summary electrophysiological data that were used to generate the figures presented in the paper are attached as supplemental tables. All other data supporting the findings of this study are available within the paper, its Supplementary information files as well as a Source data file.

Field-specific reporting

Please select the one below that is the best fit for your research. If you are not sure, read the appropriate sections before making your selection.

☒ Life sciences ☐ Behavioural & social sciences ☐ Ecological, evolutionary & environmental sciences

For a reference copy of the document with all sections, see nature.com/documents/nr-reporting-summary-flat.pdf

Life sciences study design

All studies must disclose on these points even when the disclosure is negative.

Sample size	We used sample sizes of 3 or more, to allow statistical comparison. No sample size calculation was performed as standard procedure in cellular electrophysiology experiments.
Data exclusions	If electrophysiological data were too small or cells were below standard (leaky), or data were excessively noisy, they were omitted
Replication	Data were replicated at least 3 times
Randomization	Control cells were studied along with test samples. The experimental studies and subsequent analysis were carried out by separate individuals. Randomization of allocation is not relevant to the present study
Blinding	yes, blinded during experiments

Reporting for specific materials, systems and methods

We require information from authors about some types of materials, experimental systems and methods used in many studies. Here, indicate whether each material, system or method listed is relevant to your study. If you are not sure if a list item applies to your research, read the appropriate section before selecting a response.

Materials & experimental systems

n/a	Involved in the study	
☒	☐	Antibodies
☐	☒	Eukaryotic cell lines
☒	☐	Palaeontology and archaeology
☒	☐	Animals and other organisms
☒	☐	Human research participants
☒	☐	Clinical data
☒	☐	Dual use research of concern

Methods

n/a	Involved in the study	
☒	☐	ChIP-seq
☒	☐	Flow cytometry
☒	☐	MRI-based neuroimaging

Eukaryotic cell lines

Policy information about cell lines

Cell line source(s)	All cell lines were from ATCC: tsA201, and ltk- mouse fibroblasts.
Authentication	Authentication by ATCC, these were low passage number cells. No further authentification performed
Mycoplasma contamination	cell lines not tested recently
Commonly misidentified lines (See ICLAC register)	none used
